# Economic and clinical burden of viral hepatitis in California: A population-based study with longitudinal analysis

**DOI:** 10.1371/journal.pone.0196452

**Published:** 2018-04-30

**Authors:** Haesuk Park, Donghak Jeong, Pauline Nguyen, Linda Henry, Joseph Hoang, Yoona Kim, Edward Sheen, Mindie H. Nguyen

**Affiliations:** 1 University of Florida College of Pharmacy, Pharmaceutical Outcomes & Policy, Gainesville, FL, United States of America; 2 Stanford University Medical Center, Division of Gastroenterology and Hepatology, Palo Alto, CA, United States of America; Centers for Disease Control and Prevention, UNITED STATES

## Abstract

**Background:**

Economic burden of HBV and HCV infection are trending upwards.

**Aims:**

Compare hepatitis B virus (HBV) and hepatitis C virus (HCV) related hospital admission rates, charges, mortality rates, causes of death in a US population-based study.

**Methods:**

Retrospective cohort analysis of HBV and HCV patients from the California Office of Statewide Health Planning and Development (2006–2013) database.

**Results:**

A total of 23,891 HBV and 148,229 HCV patients were identified. Across the 8-year period, the mean increase for all-cause ($1,863 vs $1,388) and liver-related hospitalization charges ($1,175 vs $675) were significantly higher for the HBV cohort compared to the HCV cohort. HBV patients had significantly higher liver-related hospital charges per person per year than HCV patients after controlling for covariates ($123,239 vs $111,837; *p* = 0.002). Compared to HCV patients, adjusted mortality hazard ratio was slightly lower in HBV patients (relative risk = 0.96; 95% CI 0.94–0.99). The major causes and places of death were different. The three major causes of death for HBV were: other malignant neoplasms (35%), cardiovascular disease/other circulatory disorders (17%), and liver-related disease (15%) whereas for HCV patients were: liver-related disease (22%), other malignant neoplasms (20%), and cardiovascular disease (16%). Regarding the place of death, 53% of HBV patients and 44% of HCV patients died in hospital inpatient, respectively.

**Conclusions:**

HBV patients incurred higher liver-related hospital charges and higher mean increase for all-cause and liver-related hospitalization charges over the 8-year period compared to HCV patients. HBV patients had slightly lower mortality rate and their major causes and places of death were noticeably different from HCV patients.

## Introduction

Chronic hepatitis B virus (HBV) and chronic hepatitis C virus (HCV) infections remain two of the leading causes of chronic liver disease, cirrhosis, and hepatocellular carcinoma (HCC) globally. HCV affects millions of people around the world and is the predominant cause of chronic liver disease, cirrhosis and HCC in the Americas, Europe, Japan, and the Middle East.[1] On the other hand, HBV is widespread throughout Asia, especially in China, and is a major cause of cirrhosis and its complications, including HCC in Asia.[2]

Both viruses are blood borne. However, the most common modes of HCV infection are through unsafe injection practices, inadequate sterilization of medical equipment, and the transfusion of unscreened blood and blood products, especially in developing countries.[3] On the other hand, though HBV is transmitted through contact with the blood or other body fluids of an infected person, the majority of cases come through perinatal transmission or in early childhood especially in more rural areas of Asia and sub-Sahara Africa despite the availability of an effective vaccine.[4]

The advent of the new direct acting antiviral agents as treatment and possible cure for HCV has changed the medical landscape concerning long-term outcomes for HCV patients. However, there is currently no such curative treatment for HBV with over 90% of those infected as infants or young children becoming chronically infected and at increased risks for cirrhosis and HCC.[5] As such, concern is raised about the burden of HCV and HBV within the United States (US). Recent studies in the US, conducted using the Nationwide Inpatient Sample (NIS), found that HBV-infected patients incurred higher inpatient resource utilization and greater inpatient mortality when compared to patients hospitalized with chronic HCV infection or alcoholic liver disease.[6,7] However, due to the cross sectional nature of the data, event-level records not patient-level recordings, these prior studies could not capture if patients required additional hospitalizations or patients died somewhere else than in a hospital. Furthermore, these studies are limited in that they reflect only liver-related utilization and deaths and represent national trends, which may not be applied to some states where high-risk populations are prevalent (e.g. Asian Americans with HBV).[6,7] Thus, additional studies with patient-level longitudinal analyses are necessary to understand the long-term clinical and economic outcomes of HBV and HCV.

Chronic liver disease and cirrhosis are among the leading causes of death in California in addition to the number of liver cancer-related deaths being much higher in California than in any other states within the United States (US).[8] As such, it is important to fully appreciate and understand the drivers of liver disease outcomes seen within the state of California as many of the other study results may not be generalizable to this populous and ethnically diverse state. Therefore, the aims of this study were to explore healthcare utilization of patients with viral hepatitis within the state of California, and given the considerable mortality associated with viral hepatitis and liver cancer in California, the secondary goal was to compare mortality rates and causes of death between HBV and HCV patients in California.

## Methods

### Data source

The Office of Statewide Health Planning and Development (OSHPD) of California maintains a database of all patient admissions to nonfederal hospitals within the state. Patients are assigned unique identifiers that allow them to be followed longitudinally in time over multiple admissions and across different hospitals until death. Therefore, we performed a retrospective analysis of the OSHPD hospital discharge database from 2006 to 2013 for all patients admitted for HBV and HCV. In California, hospitals and other inpatient healthcare facilities licensed by the state are required to submit data to the OSHPD semi-annually about all patients admitted.[9] Thus, the OSHPD databases provide almost a complete capture of all procedures within the state, omitting only those performed at federal institutions. Each hospitalization record included information on patient's demographics, expected source of payment, facility type, and hospital charges. A primary International Classification of Diseases (ICD-9) diagnosis and up to 24 secondary diagnoses listed in no particular order were also included. Individual patients were assigned a unique identification code [record linkage number (RLN)], which was derived from a complex algorithm based on the social security number of the patient and allowed for longitudinal follow up. Records for patients without a social security number were assigned a missing RLN.[10]

Mortality data were extracted from the California State Death Statistical Master file records (DSMF)—a database of death certificates for all individuals dying in and outside of California. Individuals record linkage numbers (RLN’s) were cross-linked to the DSMF via a method that was previously reported. [11] Mortality data was only available for patients who hospitalized from 2006 to 2011 with follow-up until 2013at the time of the study. The study was approved by the Institutional Review Board of the California Health and Human Services Agency and was exempted by the Stanford University Institutional Review Board.

### Study sample for all-cause hospital admission rates and charge analysis

We included all adult patients (≥18 years) who were hospitalized in California with HBV or HCV infection listed as a primary or secondary diagnosis during 2006–2013 using the ICD-9 diagnosis codes. HBV infection was identified using the following ICD-9 CM codes: 070.20, 070.30, 070.42, 070.52, and V02.61. HCV infection was identified using the following ICD-9 CM codes: 070.44, 070.54, 070.70, 070.71, and V02.62. Patients were excluded if they had a missing RLN or they had diagnosis code(s) for HBV/HCV co-infection, HIV co-infection, or other concurrent liver diseases such as alcohol liver disease, nonalcoholic fatty liver disease (NAFLD), autoimmune hepatitis, or cryptogenic chronic hepatitis. However, we performed sensitivity analyses with all HBV and HCV patients excluding HBV/HCV co-infection only.

To examine all-cause hospital related hospital rates and charges for HBV and HCV patients who were hospitalized, we classified any visit or discharge regardless a liver diagnosis as an all cause hospital analysis. For example, if a patient with HBV was admitted for a cardiovascular issue than that hospitalization was captured under all cause hospitalization and not under liver related hospitalization.

### Study sample for liver-related hospital and charge analysis

To examine liver-related hospital admissions and charges for HBV and HCV patients who were hospitalized, we classified visits and discharges as liver-related if the hospitalization involved the HBV or HCV infection, or end-stage liver disease including cirrhosis, decompensated cirrhosis (or of the clinical symptoms of decompensated cirrhosis), HCC, or liver transplant as a primary diagnosis.

### Study sample for mortality analysis

Mortality data was available for patients who were hospitalized from 2006 to 2011 but not for patients who were hospitalized from 2012–2013. Mortality data provides information on death or being alive by December 31, 2013 for those who hospitalized during 2006–2011. We included patients hospitalized with HBV or HCV infection listed as a primary or secondary diagnosis during 2006–2011 and linked to the DSMF for mortality analysis. Patients were followed up from the first hospital admission until December 31, 2013.

### Outcomes and covariates

The outcomes in this study included hospitalization rate, days in hospital, hospital charges, and mortality. The mean numbers of inpatient visits and hospital days per person per year (PPPY) were estimated for all-cause and liver-related admissions during the study period, respectively. Medical charges PPPY were also calculated for all-cause and liver-related admissions. Because hospital costs and reimbursement were not publically available, we used charges to estimate the economic burden for HBV and HCV infection. All charges were inflation (3%) adjusted to 2013 US dollars. Finally, we used Poisson regression models to estimate the adjusted relative risk of dying associated with for those diagnosed with HBV and HCV over time. Crude death rates per 1000 person-years were estimated. Reasons for death were classified as liver-related (including viral hepatitis, alcohol liver disease, hepatic decompensation, liver cancer, non-alcohol liver disease), neoplasms, drug poisoning, accidents (i.e., unintentional injury, intentional self-harm), cardiovascular disease and other circulatory disorders, nephritis, all other infections, chronic obstructive pulmonary disease, and others. Places for death were classified as hospital inpatient, patients’ residence, nursing home, hospice, and others.

Covariates in our models included demographics, insurance type, liver severity, presence of comorbidity, and degree of sickness as indicated by the Charlson Comorbidity Index (CCI). Liver severity was defined as having diagnosis of cirrhosis, decompensated cirrhosis, HCC, and liver transplant. Using the ICD-9 CM, we identified the comorbidities contained in the CCI (e.g., cardiovascular disease, chronic kidney disease, chronic obstructive pulmonary disease) and then calculated the CCI scores.

### Statistical analyses

Descriptive statistics included means (standard deviation: SD) and relative frequencies for continuous and categorical data, respectively. Individual variables were compared between HBV and HCV cohorts. Dichotomous variables were compared using Chi-square tests. Continuous variables were compared using independent t-tests and Mann-Whitney’s U-tests.

For regression analysis, we adjusted for age, gender, race, insurance type, liver severity, presence of comorbidities, and CCI scores (S1 Table). Poisson regression was used to estimate the number of hospital admissions and days in hospital. A generalized linear model (GLM) with a gamma distribution and log-link function was used to compare hospital charges. A Poisson regression model was used to measure the difference in mortality between HBV and HCV cohorts. Within the HBV and HCV cohorts, GLM and Poisson regression models were used to understand factors associated with hospital charges and mortality. All statistical analyses were 2-tailed, with an *a priori* significance level of α = 0.05. All analyses were conducted using SAS 9.4 (SAS Institute Inc., Cary, NC) and STATA version 14 (Stata Corp., College Station, TX).

## Results

### Patient characteristics

The total number of HBV patients included in the study was 23,891 while there were 148,229 HCV patients. (S1 Fig) Table 1 summarizes the baseline demographic characteristics and comorbid conditions between two cohorts. There were significant demographic differences between the two cohorts. The age range for the majority of patients in both cohorts was between 45 years and 65 years old (HBV 45% vs. HCV 65%) although more HBV patients were ≥65 years old (29% vs 16%, *p*<0.001). There were significantly more Asians in the HBV cohort (HBV 44% vs HCV 5%, *p* <0.001), significantly more HBV patients on Medicare (34% vs 28%, *p*<0.001), and significantly more HBV patients having end-stage liver disease complications such as HCC compared to HCV patients (8% vs 3%, p<0.001). Furthermore, HBV patients had slightly higher the mean CCI score (1.77 vs 1.47, *p*<0.001) but were much less likely to have a history of alcohol abuse/dependence or drug abuse/dependence as compared to HCV patients (4% vs 11%, *p*<0.001; 17% vs 43%, *p*<0.001; respectively).

**Table 1:** Characteristics of HBV and HCV patients admitted to hospital by year, US, 2006–2013.

Patient Characteristics	HBV (N = 23,891)	HCV (N = 148,229)	P-value
** Age, n (%)**			
18-<45	6344 (27)	28506 (19)	<0.001
45–65	10647 (45)	96353 (65)	
>65–75	3753 (16)	14848 (10)	
>75	3147 (13)	8522 (6)	
**Male, n (%)**	12848 (54)	90718 (61)	<0.001
**Race, n (%)**			
Asian	10440 (44)	6724 (5)	<0.001
Non-Asian (White)	9127 (38)	104123 (70)	
Non-Asian (non-White)	4324 (18)	37382 (25)	
**Insurance, n (%)**			
Medicare	8227 (34)	42081 (28)	<0.001
Medicaid	6143 (26)	42939 (29)	
Private	6829 (29)	27799 (19)	
Self-pay	869 (4)	11784 (8)	
Others	1823 (8)	23626 (16)	
**Liver severity, n (%)**			
Cirrhosis	3931 (16)	27957 (19)	<0.001
Decompensated cirrhosis	4122 (17)	23604 (16)	<0.001
Liver transplant	287 (1)	1443 (1)	<0.001
Hepatocellular carcinoma (HCC)	1900 (8)	4611 (3)	<0.001
**Charlson Comorbidity Index (CCI) score, mean (SD)**	1.77 (2.13)	1.47 (1.81)	<0.001
CCI, n (%)			
0	8884 (37)	55972 (38)	<0.001
1	4439 (19)	38441 (26)	
2	3938 (16)	21670 (15)	
3	2855 (12)	15258 (10)	
> = 4	3775 (16)	16888 (11)	
**Health and behavioral risks, n (%)**			
Alcohol abuse/dependence	1036 (4)	15703 (11)	<0.001
Drug abuse/dependence	3996 (17)	63721 (43)	<0.001

In a sensitivity analysis, when all HBV (N = 29,773) and HCV patients (N = 201,922) were included, HCV patients were significantly more likely to have concurrent alcohol liver disease (17% vs 6%, p<0.001). (S2 Table)

### Trends in hospital charges from 2006 to 2013

**Fig 1:**
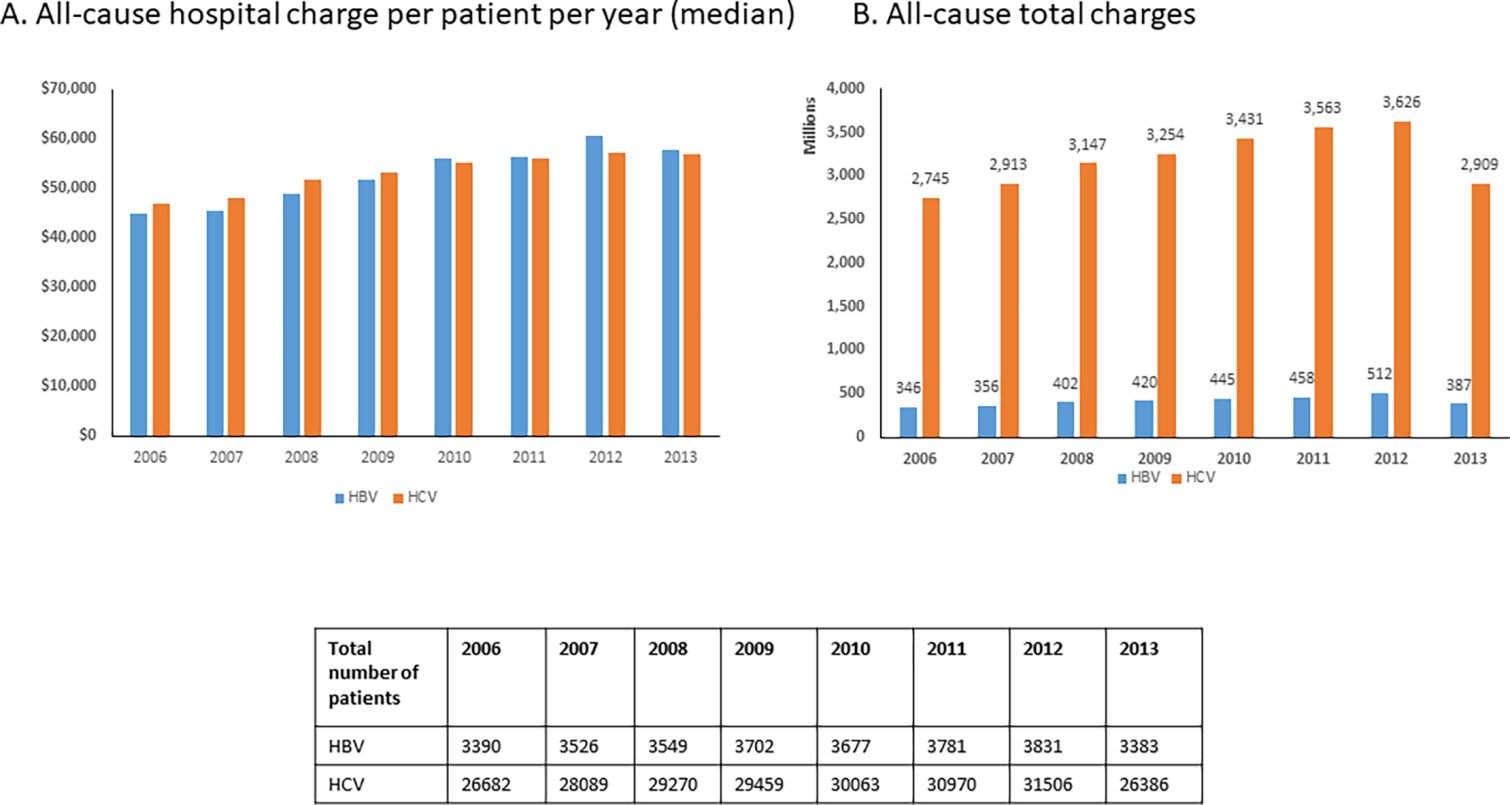
Trends in all-cause hospital charges between 2006 and 2013 in HBV and HCV patients. A. All-cause hospital charge per patient per year (median). B. All-cause total charges.

Fig 1 depicts the annual trends in median charges for care PPPY (A) and the annual trends in total charges (B) in both HBV and HCV cohorts during 2006–2013. Across the 8-year period, the median all-cause hospitalization charges increased from $44,772 to $57,811, a 5% annual increase ($1863) for the HBV cohort (*p*<0.001) and from $47,027 to $56,743, a 3% annual increase ($1388) for the HCV cohort (*p*<0.001), respectively. All-cause total charges increased 48% (from $346 million to $512 million) for HBV and 32% (from $2.7 billion to $3.6 billion) for HCV between 2006 and 2012, but decreased 24% and 20% in 2013 for the HBV and the HCV cohorts, respectively.

The median liver-related hospitalization charges increased from $49,700 to $57,927, a 2% annual increase ($1175) for the HBV cohort (*p* = 0.057) and from $46,575 to $51,299, a 1.4% annual increase ($675) for the HCV cohort (*p* <0.001), respectively. (S2 Fig) For HCV patients, liver-related total charges increased 20% from 369 million to 445 million between 2006 and 2012 but decreased 24% in 2013, whereas the total charges gradually decreased from 2006 to 2013 in the HBV cohort.

### All-cause hospital admission rates and charge analysis

HBV patients and HCV patients experienced an average of 1.27 (± 0.77) and 1.54 (±1.26) hospitalizations yearly during 2006–2013, respectively. (S3 Table). After adjusting for the covariates of demographics, insurance type, liver disease severity, and comorbidities (Table 2), the HBV cohort had an average all-cause charge of $105,223 (95% CI: $102,932-$107,515) PPPY, was significantly less than those of the HCV cohort ($112,128, 95% CI: $111,276-$112,980 PPPY; *p*<0.001).

**Table 2:** Adjusted hospital admission rates and charges per patient per year with hepatitis B or C infection, 2006–2013.

All cause hospital admission ^a^	HBV(N = 23,891)	HCV(N = 148,229)	P-value
Number of hospitalization (mean, 95% CI)	1.42 (1.41–1.44)	1.52 (1.52–1.53)	<0.001^c^
Days in hospital stay (mean, 95% CI)	11.32 (11.28–11.37)	11.32 (11.30–11.33)	0.727^c^
Hospital charges (mean, 95% CI)	$105,223 ($102,932 - $107515)	$112,128 ($111,276 - $112,980)	<0.001^d^
**Liver-related hospital admission** ^**b**^			
Number of patients	4,184	22,168	
Number of hospitalization (mean, 95% CI)	1.38 (1.34–1.42)	1.40 (1.38–1.41)	0.486^c^
Days in hospital stay (mean, 95% CI)	9.22 (9.11–9.33)	8.99 (8.86–8.93)	<0.001^c^
Hospital charges (mean, 95% CI)	$123,239 ($116,497 - $129,812)	$111,837 ($109,314- $114,360)	0.002^d^

a All cause denotes inpatient claims with an ICD-9-CM of HBV or HCV in the primary or secondary diagnosis

b "Liver-related" denotes inpatient claims with an ICD-9-CM diagnosis of HBV or HCV or end-stage liver disease in the primary diagnosis

c Poisson regression of hospital admission rates by hepatitis B or C infection controlling for demographics, insurance type, liver severity, and comorbidities

d Generalized linear model (gamma distribution with log link function) of hospital charges by hepatitis B or C virus infection controlling for demographics, insurance

### Liver-related hospital admission rates and charge analysis

Of the HBV patients admitted to the hospital, 18% (n = 4184) were admitted for a liver-related reason as their primary diagnosis while 15% (n = 22,168) of the HCV cohort hospitalized were admitted for a liver-related reason as their primary diagnosis. (S3 Table) The mean unadjusted liver-related hospital charges for patients with HBV were a ($12,993) higher than patients with HCV. After adjusting for pertinent covariates, HBV patients still had a 10% ($11,402) higher liver-related hospital charge and a 0.23 longer length of stay PPPY than the HCV cohort ($123,239 vs. $111,837, *p* = 0.002; 9.22 vs 8.99, *p<*0.001). (Table 2)

### Factors related to total hospital charges

Table 3 shows factors for total annual charges in HBV and HCV patients. For both of the HBV and HCV patients admitted to the hospital, the factors significantly related to increased annual charges were being of Asian race, having cirrhosis, decompensated cirrhosis, undergoing a liver transplant or having HCC as well as having higher CCI score. Charges related to decreased hospital charges for both HBV and HCV patients was age greater than 75 years old. Among HCV patients, male gender was associated with decreased annual hospital charges.

**Table 3:** Factors for total annual charge in patients who admitted to hospital for HBV and HCV.

	HBV (N = 23,891)		HCV (N = 148,229)	
Predictors	Mean difference in charge	*P-value*	Mean difference in charge	*P-value*
**Age**				
18-<45	Reference		Reference	
45–65	-$15,289	0.096	-$9,814	0.03
65–75	-$22,176	0.067	-$21,932	< .0001
>-75	-$64,050	< .0001	-$45,618	< .0001
**Gender**				
Female	Reference		Reference	
Male	$3,544	0.542	- $8,645	< .0001
**Race**				
Non-Asian(Non-White)	Reference		Reference	
Asian	$27,894	< .001	$16,042	0.001
Non-Asian(White)	$4,597	0.535	$3,587	0.137
**Liver Severity**				
Cirrhosis	$14,285	0.036	$12,797	< .0001
Decompensated cirrhosis	$34,549	< .0001	$36,417	< .0001
Liver transplant	$223,135	< .0001	$191,995	< .0001
Hepatocellular carcinoma	$42,632	< .0001	$66,853	< .0001
**Comorbidity**				
Charlson Comorbidity Index score				
0	Reference		Reference	
1	$7,835	0.311	$1,046	0.75
2	$42,306	< .0001	$20,315	< .0001
3	$53,182	< .0001	$38,566	< .0001
> = 4	$61,660	< .0001	$56,386	< .0001

### Mortality analysis

Table 4 shows morality rates and causes of death for inpatient HBV and HCV patients. We identified 18,437 HBV patients and 119,020 HCV patients who were hospitalized during 2006–2011 with mortality information on death or being alive by December 31, 2013. Of these, 25% of the HBV cohort (n = 5201) died giving a crude mortality rate of 71.97 events per 1000 person years (mean follow-up + 3.92 years). Overall, the three major causes of death for patients with HBV and with a known cause of death were other malignant neoplasms (35%), followed by cardiovascular disease/other circulatory disorders (17%), and then liver-related disease (15%).

**Table 4:** Mortality rates and causes of death for inpatient HBV and HCV patients.

	HBV (N = 18,437)	HCV (N = 119,020)
Mean follow-up (mean) years	3.92	4.10
Crude mortality rate—events per 1000 person- years	71.97 per 1000 person-years (5201/72277)	73.30 per 1000 person-years (35473/487600)
Adjusted hazard ratio (HR, 95% CI)	0.96 (0.94–0.99)	Reference
**Cause of death, n (%)**		
Liver-related disease: alcohol liver disease, hepatic decompensation, HCV, HBV, other viral hepatitis, liver cancer, non alcohol liver disease	759 (15)	7749 (22)
Neoplasms: malignant lymphoid neoplasms	222 (4)	586 (2)
Other malignant neoplasms	1837 (35)	6919 (20)
Cardiovascular disease and other circulatory disorders	861 (17)	5620 (16)
All other infections (except viral hepatitis)	187 (4)	1252 (4)
Accidents: unintentional injury, intentional self-harm	76 (2)	872 (3)
Nephritis, nephrotic syndrome, and chronic kidney disease	113 (2)	565 (2)
Chronic obstructive pulmonary disease and pneumonia	101 (2)	894 (3)
Drug poisoning and other substance use disorder	71 (1)	1829 (6)
Diabetes mellitus	32 (0.6)	210 (0.6)
Hemorrhage	21 (0.4)	185 (0.5)
Unknown causes of mortality	13 (0.2)	158 (0.4)
Other	660 (13)	4221 (12)
Missing data	248 (5)	4413 (12)

For the HCV cohort, 30% (n = 35,473) of the patients died giving a crude mortality rate of 73.30 events per 1000 person- years (mean follow-up +4.10 years). The three major causes of death for HCV patients with a known cause of death were liver-related disease (22%), other malignant neoplasms (20%), and cardiovascular disease/other circulatory disorders (16%).

Regarding the place of death, 53% of HBV patients and 44% of HCV patients died in as a hospital inpatient followed by patients’ residence (23% HBV vs 22% HCV) and lastly, the nursing home/convalescent home (12% HBV vs 10% HCV). (S4 Table) Overall, the inpatient mortality rate was higher for the HBV cohort compared to the HCV cohort (HBV: 38 per 1000 person years vs HCV: 32 per 1000 person years: data not shown).

After adjusting for covariates, HBV patients had slightly lower mortality rate compared to HCV patients (adjusted relative risk (RR) = 0.96; 95% CI 0.94–0.99). S5 Table shows factors associated with mortality in HBV and HCV patients. In the HBV cohort, patients were almost two times as likely to die if they were age 75 or older (RR: 2.56). In addition, they were 18% more likely to die if there were male (RR: 1.18), 48% and 22% more likely if they had decompensated cirrhosis (RR: 1.48) or HCC (RR: 1.22) and 6 times more likely with a CCI score > = 4 (RR: 6.41). Meanwhile, being Asian and liver transplant recipients were all associated with a decreased risk of death. The factors were similar for the HCV cohort.

## Discussion

In this large statewide inpatient study, we compared HBV patients and HCV patients on demographics, in-hospital resource utilization, and mortality. We found, as others, that HBV and HCV patients incur significant inpatient charges which trended up over time through 2012, especially within the HBV population. [1–6] However, the numbers of HBV and HCV patients admitted to hospitals decreased in 2013 resulting in significant decreases in the all-cause and liver-related total changes for 2013. These decreases are explained by an overall decrease in statewide rates of newly reported HBV and HCV infections between 2009 and 2013 according to the report, “Chronic Hepatitis B and C in California 2013 Executive Summary”. [12, 13] These downward trends are encouraging in that preventive and treatment efforts that were implemented under Institute of Medicine’s report in 2010 entitled, “Hepatitis and Liver Cancer: A National Strategy for Prevention and Control of Hepatitis B and C” [14] may, in fact, be working; however, this drop will need to be followed over time to determine if this is a continuing trend especially as new treatments for HCV and HBV are introduced for the cure and care of patients.

Our HBV cohort was older, sicker, more likely to have end- stage liver disease (HCC) and to die in the hospital than HCV patients. Specifically, the major cause of death for the HBV cohort was other malignancy which accounted for 35% for the deaths followed by cardiovascular disease (17%). We found that the 53% of HBV patients died in hospital inpatient. Our study documents HBV patients incurred longer hospital length of stays and higher hospital charges for liver related hospitalizations compared to HCV patients. This is likely due to the higher liver severity with HBV (e.g., HCC). These findings are similar to other studies which found significantly higher resource utilization for their HBV patients and that they are more likely to die from other cancers such as stomach, lung and esophageal cancer as well as non-Hodgkin’s lymphoma as a result of the interplay of HBV and the immune system which allows HBV patients to be more susceptible to other diseases such as cancers. [15–20] On the other hand, we found that Asian patients were less likely to die. We suggest that this result may be due to better adherence to the HCC screening guidelines (those at risk for HCC be screened every six months) resulting in earlier and potentially curative treatment amongst this cohort of patients. [21] Other studies also found that among HCC patients, Asian patients had higher survival rates although none have been able to discern a cause. [22–24]

Another significant finding of our study was that HBV patients had slightly lower mortality compared to HCV patients when other places of death were included whereas the previous studies using NIS concluded that HBV patients incurred higher inpatient mortality compared to HCV patients. [6,7] We identified that approximately half the patients died outside the hospital (47% in HBV and 56% in HCV patients) with the primary place outside the hospital being the patients’ residence. However, an interesting finding was that more HCV patients than HBV patients (17% vs 7%, respectively) had an unknown place of death. This finding may be associated with higher health risk behaviors (e.g., drug/alcohol abuse) seen within the HCV cohort and may also explain their higher missing data (12%) and drug poisoning (6%) for cause of death—again suggesting high-risk health behaviors as reasons for the cause of death. Such conjecture would be in line with a study conducted by Amin et al who found that the risk of dying from drug-related causes was significantly greater than from liver-related reasons for people with HCV especially younger female patients. [25] In a sensitivity analysis, when all HBV and HCV patients were included, we also found that HBV patients had lower mortality rates compared to HCV patients.

Although the growing healthcare burden associated with HCV infection is gaining attention due to its high prevalence and new direct-acting antivirals available, the prevalence and severity of HBV-related hospitalization and mortality are under-recognized. With ongoing mitigation from countries of high HBV endemicity, the number of individuals with chronic HBV in the United States including California is expected to continue to grow. [26–27] However, approximately two-thirds of Asian Americans who are chronically infected with HBV are unaware that they are infected in California. [28] This may impose high clinical and economic burden to patients and health systems in California because chronic HBV infection are unmanaged and may diagnosed after advanced liver disease has already developed. Our study suggests that despite the fact that HBV infection is much less frequent than for HCV infection in the U.S., HBV infection continues to be a significant burden.

Our study has a few limitations. The first was that the data were administrative which were reliant on accurate coding. However, since the data were from a single state, there was more consistency in the coding. Secondly, as a result of the lack of information about the timing of the initial diagnosis, outpatient visits, and treatment (e.g., antiviral therapy) we were unable to control for the timing of these variables on patient related outcomes which can alter patient outcomes. Thirdly, since our sample sizes were very large small, differences in outcomes may become statistically significant but may not be clinically significant. In our discussion, we highlighted the findings that we felt were both statistically and clinically significant. Fourthly, the OSHPD databases provide almost a complete capture of all procedures within the state (96%); however, approximately 4% of the CA adult population were veterans who received their care at the Veterans Administration hospitals so were not included in this analysis. [10] Finally, the study period ends before the introduction of the new direct acting antiviral agents for the treatment of HCV as well as different combination drugs for HBV; however, as noted, our study found a decline in the number of new HBV and HCV cases reported to the state of California suggesting that as these new treatment regimens are used, this trend will continue but will need further study.

Despite these limitations, this study has several notable features. First, our study contained a much higher proportion of Asian patients in our HBV study population compared to another study using the national inpatient data (44% vs. 11%, respectively) [6] making our results more generalizable to Asian patients with chronic HBV infection. Second, we used hospitalization diagnosis codings for HBV and HCV as either primary or one of up to 24 secondary diagnoses to ensure that all cause hospitalizations were included because individuals with HBV and HCV infection have a higher rate of morbidity and death due to all causes. Third, the OSHPD data used unique identifiers which allowed for our longitudinal analyses and cross-linkage with death records for the mortality analysis. [10] We were also able to comprehensively examine the causes and places of death in HBV and HCV patients, which was helpful in understanding of their death. Also, as confirmed by the state of California, the dataset used in this study was a complete dataset so our results for 2013 are final allowing our results as a potential comparison source for new treatment that have or may come to market after 2013.

In conclusion, patients with HBV and HCV require extensive use of inpatient hospital resources. HBV patients incurred higher liver-related hospital charges and longer hospital stays. PPPY charges for hospitalization increased for both cohorts up to 2013. Although HBV patients had slightly lower mortality, approximately 15% of the HBV patients and 22% of the HCV patients died from a liver-related cause whereas over 39% of the HBV patients and 22% of the HCV patients died from other cancers. As HCV cure is available, further studies are needed to evaluate how new DAAs will potentially reduce the economic burden and mortality for HCV patients. There is currently no cure for HBV but several drug regimens are finding success in delaying the progression of liver disease. Further effort is needed to screen, diagnose, and link patients with viral hepatitis to care before the development of major complications with associated hospitalization and premature death.

## Supporting information

S1 Fig Flow diagram of number of patients included in HBV and HCV cohorts. (TIF)

S2 Fig Unadjusted hospital admission rates and charges per person per year in HBV and HCV patients, 2006–2013. (TIF)

S1 Table List of covariates adjusted in regression models. (DOCX)

S2 Table Characteristics of all HBV and HCV patients admitted to hospital by year, US, 2006–2013. (DOCX)

S3 Table Trend in liver-related hospital charges between 2006 and 2013 in HBV and HCV patients. (DOCX)

S4 Table Place of death for inpatient HBV and HCV patients. (DOCX)

S5 Table Factors associated with mortality in inpatient HBV and HCV patients. (DOCX)

## References

[1] World Health Organization (WHO). Hepatitis C. 2016. Available at http://www.who.int/mediacentre/factsheets/fs164/en/. Accessed June 10, 2017.

[2] Hajarizadeh B, Grebely J, Dore GJ. Epidemiology and natural history of HCV infection. Nat Rev Gastroenterol Hepatol. 2013:10(9):553–562. doi: 10.1038/nrgastro.2013.107 23817321 10.1038/nrgastro.2013.107

[3] Nguyen LH, Nguyen MH. Systematic review: Asian patients with chronic hepatitis C infection. Aliment Pharmacol Ther. 2013:37(10):921–936. doi: 10.1111/apt.12300 23557103 10.1111/apt.12300

[4] World Health Organization (WHO). Hepatitis B. 2016. Available at http://www.who.int/mediacentre/factsheets/fs204/en/. Accessed June 10, 2017.

[5] Chen CJ, Yang HI. Natural history of chronic hepatitis B REVEALed. J Gastroenterol Hepatolo. 2011:26(4):628–638. 10.1111/j.1440-1746.2011.06695.x 21323729

[6] Cholankeril G, Perumpail RB, Hu M, Skowron G, Younossi ZM, Ahmed A. Chronic Hepatitis B Is Associated with Higher Inpatient Resource Utilization and Mortality Versus Chronic Hepatitis C. Dig Dis Sci. 2016:61(9):2505–2515. doi: 10.1007/s10620-016-4160-z 27084385 10.1007/s10620-016-4160-z

[7] Rajbhandari R, Danford CJ, Chung RT, Ananthakrishnan AN. HBV infection is associated with greater mortality in hospitalised patients compared to HCV infection or alcoholic liver disease. Aliment Pharmacol Ther. 2015:41(10):928–938. doi: 10.1111/apt.13162 25786514 10.1111/apt.13162

[8] Seigel R, Miller K, Jemal A. Cancer statistics 2017. CA Cancer J Clin. 2017: 67(1): 7–30 10.3322/caac.21387 28055103

[9] Office of Statewide Health Planning and Development. California Inpatient Data Reporting Manual, Medical Information Reporting for California. 2017. Avilable at http://www.oshpd.ca.gov/hid/mircal/IPManual.html. Accessed June 10, 2017.

[10] Sie L, Gatto NM, Bancroft E. Hospitalizations due to hepatitis C in Los Angeles County, 2007–2009: case characteristics and factors associated with mortality. J Viral Hepat. 2013:20(9):628–637. 10.1111/jvh.12086 23910647

[11] Zingmond DS, Ye Z, Ettner SL, Liu H. Linking hospital discharge and death records—accuracy and sources of bias. J Clin Epidemiol. 2004:57(1):21–29. doi: 10.1016/S0895-4356(03)00250-6 15019007 10.1016/S0895-4356(03)00250-6

[12] California Department of Public Health (COPH). Chronic Hepatitis B Infections in California Surveillance Report, 2013: Executive Summary. 2016. Available at https://www.cdph.ca.gov/programs/Documents/ChronicHBV_SurvRpt_ExecSum.pdf.

[13] California Department of Public Health (COPH). Chronic Hepatitis C Infections in California Surveillance Report, 2013: Executive Summary. 2016. Available at https://www.cdph.ca.gov/programs/Documents/ChronicHCV_SurvRpt_ExecSum.pdf.

[14] Institue of Medicine. Hepatitis and liver cancer: A national strategy for prevention and control of hepatitis B and C Washington, DC: The National Academies Press https://doi.org/10.17226/12793. 25032367

[15] Duberg AS, Torner A, Davidsdottir L, Aleman S, Blaxhult A, Svensson A, et al Cause of death in individuals with chronic HBV and/or HCV infection, a nationwide community-based register study. J Viral Hepat. 2008:15(7):538–550. doi: 10.1111/j.1365-2893.2008.00982.x 18397223 10.1111/j.1365-2893.2008.00982.x

[16] Walter SR, Thein HH, Amin J, Gidding HF, Ward K, Law MG, et al Trends in mortality after diagnosis of hepatitis B or C infection: 1992–2006. J Hepatol. 2011:54(5):879–886. 10.1016/j.jhep.2010.08.035 21145812

[17] Montuclard C, Hamza S, Rollot F, Evrard P, Faivre J, Hilton P, et al Causes of death in people with chronic HBV infection: A population-based cohort study. J Hepatol. 2015:62(6):1265–1271. doi: 10.1016/j.jhep.2015.01.020 25625233 10.1016/j.jhep.2015.01.020

[18] Gidding HF, Dore GJ, Amin J, Law MG. Trends in all cause and viral liver disease-related hospitalizations in people with hepatitis B or C: a population-based linkage study. BMC Public Health. 2011:11:52 doi: 10.1186/1471-2458-11-52 21261993 10.1186/1471-2458-11-52 PMC3039587

[19] Szpakowski JL, Tucker LY. Causes of death in patients with hepatitis B: a natural history cohort study in the United States. Hepatology. 2013:58(1):21–30. doi: 10.1002/hep.26110 23080403 10.1002/hep.26110

[20] Chen G, Lin W, Shen F, Iloeje UH, London WT, Evans A. Chronic hepatitis B virus infection and mortality from non-liver causes: results from the Haimen City cohort study. Int J Epidemiol. 2005:34(1):132–137. doi: 10.1093/ije/dyh339 15659459 10.1093/ije/dyh339

[21] Tran SA, Le A, Zhao C, Hoang J, Yasukawa LA, Weber S, et al Rate of hepatocellular carcinoma surveillance remains low for a large real-life cohort of patients with hepatitis C cirrhosis. BMJ Open Gastroenterology 2018: 5(1) http://dx.doi.org/10.1136/bmjgast-2017-000192 10.1136/bmjgast-2017-000192 PMC5873543 29607053

[22] Devaki P, Wong RJ, Marupakula V, Nangia S, Nguyen L, Ditah ID, et al Approximately one-half of patients with early-stage hepatocellular carcinoma meeting Milan criteria did not receive local tumor destructive or curative surgery in the post-MELD exception era. Cancer 2014;120(11):1725–1732. doi: 10.1002/cncr.28639 24590359 10.1002/cncr.28639

[23] Yip B, Wantuck JM, Kim LH, Wong RJ, Ahmed A, Garcia G, et al Clinical presentation and survival of Asian and non-Asian Patients with HCV-related hepatocellular carcinoma. Dig Dis Sci. 2014;59:192–200. doi: 10.1007/s10620-013-2948-7 24282055 10.1007/s10620-013-2948-7

[24] Kim N, Nguyen P, Dang H, Kumari R, Garcia G, Esquivel CO, et al Temporal trends in disease presentation and survival of patients with hepatocellular carcinoma: a real-world experience from 1998–2015. Cancer 2018 doi: 10.1002/cncr.31373[In press]. 29624631 10.1002/cncr.31373

[25] Amin J, Law MG, Bartlett M, Kaldor JM, Dore GJ. Causes of death after diagnosis of hepatitis B or hepatitis C infection: a large community-based linkage study. Lancet. 2006:368 (9539): 938–945. doi: 10.1016/S0140-6736(06)69374-4 16962883 10.1016/S0140-6736(06)69374-4

[26] Mitchell T, Armstrong G, Hu D, Wasley A, Painter J. The increasing burden of imported chronic hepatitis B- United States, 1974–2008. PLOS One. 2013:8(3): doi: 10.1371/annotation/7f73ed17-709e-4d7f-9aae-aab1f4a34985 10.1371/annotation/7f73ed17-709e-4d7f-9aae-aab1f4a34985 PMC5769802 29294478

[27] Kowdley K, Wang C, Welch S, Roberts H, Brosgart C. Prevalence of chronic hepatitis B among foreign-born persons living in the United States by country of origin. Hepatology 2012”56:422–433. doi: 10.1002/hep.24804 22105832 10.1002/hep.24804

[28] Lin S, Chang E, So S. Why we should routinely screen Asian American adults for hepatitis B: a cross-sectional study of Asians in California. Hepatology. 2007:46(4):1034–1040. doi: 10.1002/hep.21784 17654490 10.1002/hep.21784

