# Correction: Economic and clinical burden of viral hepatitis in California: A population-based study with longitudinal analysis

**DOI:** 10.1371/journal.pone.0209715

**Published:** 2018-12-19

**Authors:** Haesuk Park, Donghak Jeong, Pauline Nguyen, Linda Henry, Joseph Hoang, Yoona Kim, Edward Sheen, Mindie H. Nguyen

The Data Availability statement for this paper is incorrect. The correct statement is: There are ethical and legal restrictions on sharing the data set due to patient privacy. Therefore, legal restrictions related to agreements made with the Office of Statewide Health Planning and Development (OSHPD) of California (https://www.oshpd.ca.gov) are in place to prevent a breach of contract. Qualifying researchers may apply to access the data by contacting Office of Statewide Health Planning and Development (OSHPD) of California, Information Services Division (dataandreports@oshpd.ca.gov).
