# Supplementary figures and images for: Economic and clinical burden of viral hepatitis in California: A population-based study with longitudinal analysis
Source: PLoS One. 2018 Apr 30;13(4):e0196452. doi: 10.1371/journal.pone.0196452 (PMC5927421; doi:10.1371/journal.pone.0196452)

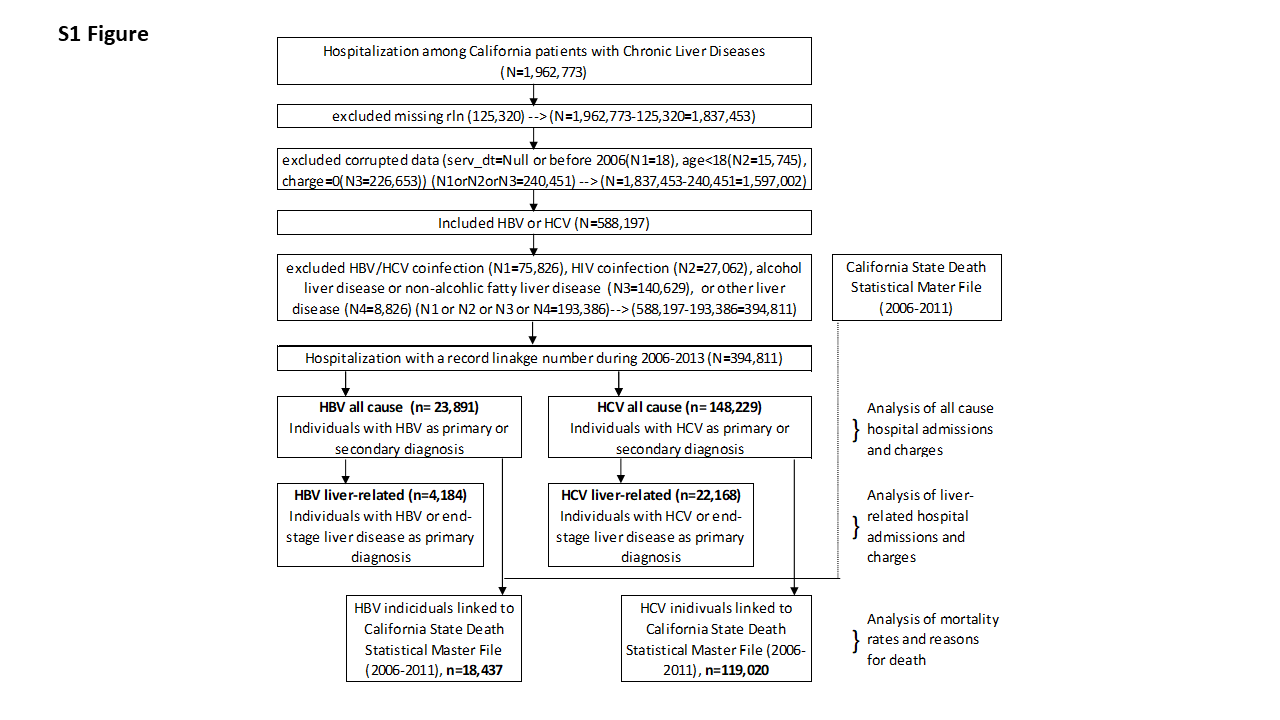

Supplement: S1 Fig — (TIF) [file pone.0196452.s001.TIF]

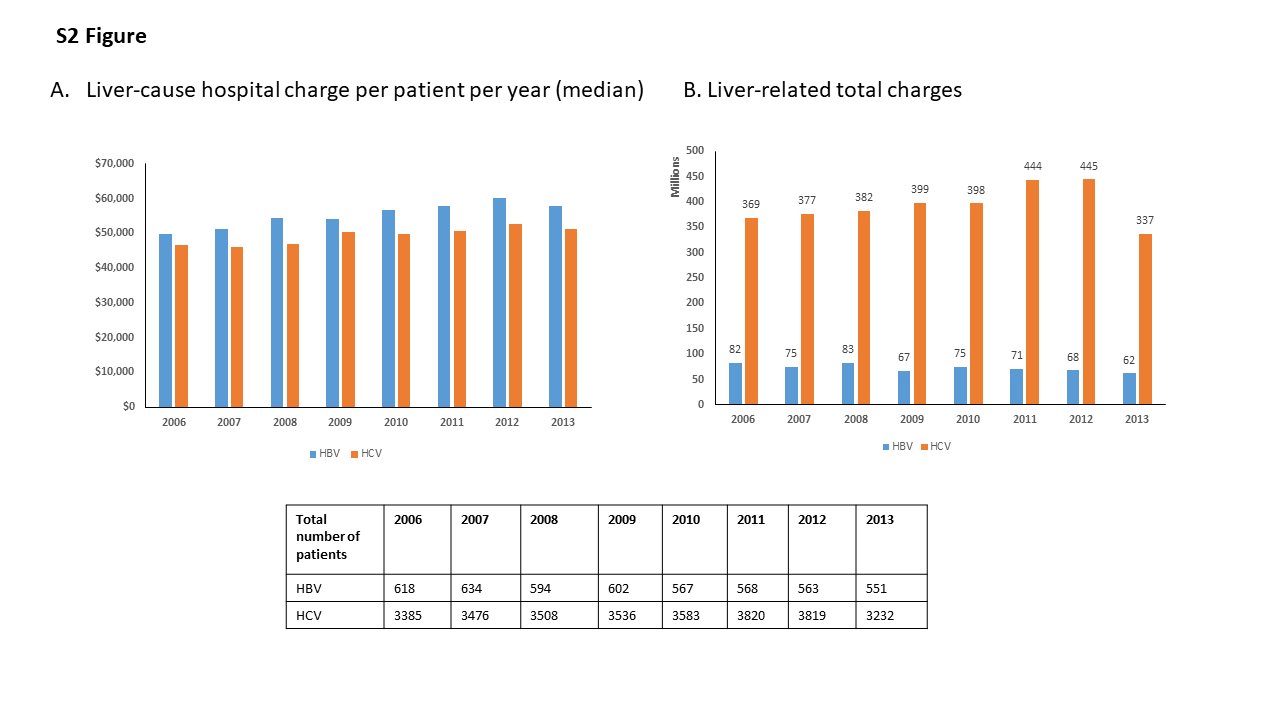

Supplement: S2 Fig — (TIF) [file pone.0196452.s002.TIF]
