# Supplementary material for: Economic and clinical burden of viral hepatitis in California: A population-based study with longitudinal analysis
Source: PLoS One. 2018 Apr 30;13(4):e0196452. doi: 10.1371/journal.pone.0196452 (PMC5927421; doi:10.1371/journal.pone.0196452)
Supplement: S1 Table — (DOCX) [file pone.0196452.s003.docx]

**S1 Table. List of covariates adjusted in regression models**

| Demographics | Age |
| --- | --- |
|  | Gender |
|  | Race |
|  | Insurance |
| Liver severity | Compensated cirrhosis |
|  | Decompensated cirrhosis |
|  | Liver transplant |
|  | Hepatocellular carcinoma |
| Comorbidity | Charlson comorbidity index (CCI) socre |
|  | Cardiovascular diseae |
|  | Diabetes |
|  | Hyperlipidemia |
|  | Hypertension |
|  | Chronic kidney disease |
|  | Chronic obstructive pulmonary disease |
| Health and behavioral risks | Alcohol abuse |
|  | Drug abuse |
