# Supplementary material for: Economic and clinical burden of viral hepatitis in California: A population-based study with longitudinal analysis
Source: PLoS One. 2018 Apr 30;13(4):e0196452. doi: 10.1371/journal.pone.0196452 (PMC5927421; doi:10.1371/journal.pone.0196452)
Supplement: S2 Table — (DOCX) [file pone.0196452.s004.docx]

| **S2 Table. Characteristics of all HBV and HCV patients admitted to hospital by year, US, 2006-2013**   \| Patient Characteristics \| **HBV**  **(N= 29,773)** \| **HCV**  **(N=201,922)** \| P-value \| \| --- \| --- \| --- \| --- \| \| **Age, n (%)** \| \| \| \| \| 18-<45 \| 8657 (29) \| 40682 (20) \| <0.001 \| \| 45-65 \| 13360 (45) \| 132021 (65) \| \| >65-75 \| 4360 (15) \| 18944 (9) \| \| >75 \| 3396 (11) \| 10275 (5) \| \| **Male, n (%)** \| 16574 (56) \| 131296 (65) \| <0.001 \| \| **Race, n (%)** \| \| \| \| \| Asian \| 12346 (42) \| 9108 (5) \| <0.001 \| \| Non-Asian (White) \| 11466 (39) \| 139721 (69) \| \| Non-Asian (non-White) \| 5961 (20) \| 53093 (26) \| \| **Insurance, n (%)** \| \| \| \| \| Medicare \| 9273 (31) \| 51009 (25) \| <0.001 \| \| Medicaid \| 8123 (27) \| 57449 (29) \| \| Private \| 8288 (28) \| 38461 (19) \| \| Self-pay \| 1572 (5) \| 16972 (8) \| \| Others \| 2517 (9) \| 38031 (19) \| \| **Liver severity, n (%)** \| \| \| \| \| Cirrhosis \| 4593 (15) \| 34628 (17) \| <0.001 \| \| Decompensated cirrhosis \| 7256 (24) \| 53018 (26) \| <0.001 \| \| Liver transplant \| 290 (1) \| 1508 (1) \| 0.0011 \| \| Hepatocellular carcinoma (HCC) \| 2277 (8) \| 6856 (3) \| <0.001 \| \| **Charlson Comorbidity Index (CCI) score, mean (SD)** \| 2.43 (2.78) \| 2.40 (2.55) \| 0.0995 \| \| CCI, n (%) 0 \| 10585 (36) \| 72314 (36) \| <0.001 \| \| 1 \| 5111 (17) \| 51625 (26) \| \| 2 \| 4466 (15) \| 28860 (14) \| \| 3 \| 3465 (12) \| 23668 (12) \| \| >=4 \| 6146 (21) \| 25455 (13) \| \| **Health and behavioral risks, n (%)** \| \| \| \| \| Alcohol abuse/dependence \| 2714 (9) \| 27059 (13) \| <0.001 \| \| Drug abuse/dependence \| 5075 (17) \| 84905 (42) \| <0.001 \| \| **Concurrent liver disease** \|  \|  \|  \| \| Alcohol liver disease \| 1790 (6) \| 33380 (17) \| <0.001 \| \| Nonalcoholic fatty liver disease (NAFLD) \| 623(2) \| 5047 (3) \| <0.001 \| \| Other liver disease (autoimmune hepatitis, hemochromatosis, Wilson’s disease, biliary cirrhosis) \| 390 (1) \| 1590 (1) \| <0.001 \| \| **HIV/AIDS** \| 1704 (6) \| 7492 (4) \| <0.001 \| |
| --- | --- | --- | --- | --- | --- | --- | --- | --- | --- | --- | --- | --- | --- | --- | --- | --- | --- | --- | --- | --- | --- | --- | --- | --- | --- | --- | --- | --- | --- | --- | --- | --- | --- | --- | --- | --- | --- | --- | --- | --- | --- | --- | --- | --- | --- | --- | --- | --- | --- | --- | --- | --- | --- | --- | --- | --- | --- | --- | --- | --- | --- | --- | --- | --- | --- | --- | --- | --- | --- | --- | --- | --- | --- | --- | --- | --- | --- | --- | --- | --- | --- | --- | --- | --- | --- | --- | --- | --- | --- | --- | --- | --- | --- | --- | --- | --- | --- | --- | --- | --- | --- | --- | --- | --- | --- | --- | --- | --- | --- | --- | --- | --- | --- | --- | --- | --- | --- | --- | --- | --- | --- | --- | --- | --- | --- | --- | --- | --- | --- | --- | --- |
