# Supplementary material for: Economic and clinical burden of viral hepatitis in California: A population-based study with longitudinal analysis
Source: PLoS One. 2018 Apr 30;13(4):e0196452. doi: 10.1371/journal.pone.0196452 (PMC5927421; doi:10.1371/journal.pone.0196452)
Supplement: S3 Table — (DOCX) [file pone.0196452.s005.docx]

**S3 Table. Unadjusted Hospital admission rates and charges per patient per year with hepatitis B or C infection, 2006-2013**

| **All cause hospital admission ^a^** | **HBV (n=23,891)** | **HCV (n=148,229)** | **P-value** |
| --- | --- | --- | --- |
| Number of hospitalization, mean (SD) | 1.27 (0.77) | 1.54 (1.26) | <0.001 |
| Hospital charges, mean (SD) | $115,294 ($216,777) | $110,088 ($196,106) | <0.001 |
| Days in hospital stay, mean (SD) | 9.87 (20.13) | 11.49 (25.91) | <.0001 |
| **Liver-related hospital admission ^b^** |  |  |  |
| Number of patients | 4,184 | 22,168 |  |
| Number of hospitalization, mean (SD) | 1.24 (0.69) | 1.42 (1.02) | <.0001 |
| Hospital charges, mean (SD) | $124,075 ($232,857) | $111,082 ($232,586) | 0.913 |
| Days in hospital stay, mean (SD) | 8.27 (10.15) | 9.05 (14.00) | <0.001 |

a "All cause" denotes inpatient claims with an ICD-9-CM of HBV or HCV in the primary or secondary diagnosis

b "Liver-related" denotes inpatient claims with an ICD-9-CM diagnosis of HBV or HCV or end-stage liver disease in the primary diagnosis
