# Supplementary material for: Economic and clinical burden of viral hepatitis in California: A population-based study with longitudinal analysis
Source: PLoS One. 2018 Apr 30;13(4):e0196452. doi: 10.1371/journal.pone.0196452 (PMC5927421; doi:10.1371/journal.pone.0196452)
Supplement: S4 Table — (DOCX) [file pone.0196452.s006.docx]

**S4 Table. Place of death for inpatient HBV and HCV patients**

| **Places of death, n (%)** | **HBV (n=18,437)** | **HCV (n=119,020)** |
| --- | --- | --- |
| Hospital inpatient | 2733 (53) | 15608 (44) |
| Patients’ residence | 1175 (23) | 7836 (22) |
| Nursing home/convalescent home | 626 (12) | 3478 (10) |
| Hospital outpatient/emergency room | 209 (4) | 1826 (5) |
| Hospice | 92 (2) | 728 (2) |
| Others/unknown | 366 (7) | 5997 (17) |
