# Supplementary material for: Economic and clinical burden of viral hepatitis in California: A population-based study with longitudinal analysis
Source: PLoS One. 2018 Apr 30;13(4):e0196452. doi: 10.1371/journal.pone.0196452 (PMC5927421; doi:10.1371/journal.pone.0196452)
Supplement: S5 Table — (DOCX) [file pone.0196452.s007.docx]

**S5 Table. Factors associated with mortality in inpatient HBV and HCV patients, 2006-2011**

|  | **HBV (N=18,437)** | **HCV (N=119,020)** |
| --- | --- | --- |
| **Covariates** | **Relative risk** | **Relative risk** |
| **Age** |  |  |
| 18-<45 | Reference | |
| 45-65 | 1.67* | 1.47* |
| 65-75 | 1.91* | 1.83* |
| >-75 | 2.56* | 2.48* |
| **Gender** |  |  |
| Female | Reference | |
| Male | 1.18* | 1.15* |
| **Race** |  |  |
| Asian | 0.87* | 0.93* |
| Non-Asian(White) | 1.05 | 1.03* |
| Non-Asian(Non-White) | Reference | |
| **Liver Severity** |  |  |
| Cirrhosis | 1.02 | 1.15* |
| Decompensated cirrhosis | 1.48* | 1.40* |
| Liver Transplant | 0.75* | 0.76* |
| Hepatocellular carcinoma | 1.22* | 1.13* |
| **Comorbidity** |  |  |
| Charlson Comorbidity Index score | | |
| 0 | Reference | |
| 1 | 2.21* | 1.78* |
| 2 | 3.91* | 2.69* |
| 3 | 4.42* | 3.19* |
| >=4 | 6.41* | 4.19* |
| Presence of chronic disease | | |
| Cardiovascular disease | 0.98 | 1.00 |
| Chronic kidney disease | 0.95 | 1.02 |
| Chronic obstructive pulmonary disease | 1.08* | 1.00 |

*indicates that P value less than 0.05.
